# Breaking the Balance: Baseline Oxidative Stress and DNA Repair Capacity in Multiple Myeloma Therapy

**DOI:** 10.3390/cancers18121995

**Published:** 2026-06-19

**Authors:** Panagiotis Malamos, Elisavet Deligianni, Konstantinos Voutetakis, Konstantinos Koutoulogenis, Olga Papadodima, Evangelos Terpos, Vassilis L. Souliotis

**Affiliations:** 1 Institute of Chemical Biology, National Hellenic Research Foundation, 116 35 Athens, Greece; pmalamos@eie.gr (P.M.); edelig@eie.gr (E.D.); kvoutet@eie.gr (K.V.); opapadod@eie.gr (O.P.); 2 Department of Nutrition and Dietetics, School of Health Science and Education, Harokopio University, 176 76 Athens, Greece; konkout@hua.gr; 3 Department of Clinical Therapeutics, National and Kapodistrian University of Athens, 115 28 Athens, Greece; eterpos@med.uoa.gr

**Keywords:** multiple myeloma (MM), DNA damage response (DDR), melphalan, redox status, apurinic/apyrimidinic (AP, abasic) sites, nucleotide excision repair (NER), double strand breaks repair (DSB/R), baseline DNA damage, chromatin condensation, apoptotic sensitivity

## Abstract

Background/Objectives: Disruption of cellular redox balance and DNA damage response (DDR) signals represents a key driver of cancer development, influencing tumor progression and therapeutic response. Here, we investigated the interplay between DDR-related parameters and oxidative stress in relation to treatment response in patients with multiple myeloma (MM). Methods: Oxidative stress and DDR signals were evaluated in primary cells, including peripheral blood mononuclear cells (PBMCs) and bone marrow plasma cells (BMPCs), collected at diagnosis from MM patients who were subsequently classified as responders (*n* = 35) or non-responders (*n* = 41) to melphalan-based therapy. Results: PBMCs and BMPCs from non-responders exhibited a distinct biological profile characterized by lower baseline DNA damage, reduced oxidative stress, increased nucleotide excision repair and double-strand break repair capacity, and reduced apoptotic sensitivity compared with responders (all *p* < 0.001). In addition, non-responders displayed increased chromatin relaxation. Differential gene expression patterns involving DDR-related pathways further distinguished BMPCs between the two clinical outcome groups. Conclusions: Collectively, these findings indicate that alterations in oxidative stress and DDR signals play a crucial role in determining response to melphalan-based therapy in MM. The identification of these molecular alterations in an easily accessible tissue, such as peripheral blood, underscores their potential clinical relevance and warrants further validation.

## Simple Summary

Multiple myeloma is a hematological malignancy characterized by considerable variability in patients’ responses to treatment. One of the key determinants of therapeutic outcome is the DNA damage response, a complex cellular network responsible for detecting and repairing genomic damage. In this study, we investigated the relationship between DNA repair capacity, oxidative stress, and response to melphalan-based therapy in patients with multiple myeloma. We analyzed malignant plasma cells from the bone marrow as well as peripheral blood mononuclear cells. We observed consistent differences between patients who responded to treatment and those who did not, including variations in DNA damage levels, oxidative stress, DNA repair activity, and apoptotic sensitivity. Notably, similar patterns were detected in both tumor and blood cells, suggesting that systemic biological features may reflect disease behavior. These findings provide insight into the biological mechanisms associated with treatment response in multiple myeloma and support further investigation of their potential clinical relevance.

## 1. Introduction

Multiple myeloma (MM) is a malignant plasma cell disorder that accounts for approximately 1% of all cancers and nearly 10% of hematologic malignancies [1]. The disease develops through a multistep process beginning with the premalignant condition monoclonal gammopathy of undetermined significance (MGUS), characterized by the production of a monoclonal immunoglobulin (M protein) by clonal plasma cells. In a subset of patients, MGUS progresses to an intermediate stage known as smoldering multiple myeloma (SMM) [2]. During the first five years following diagnosis, SMM carries a progression risk of up to 10% per year, decreasing to about 3% annually for the next five years and then to approximately 1% per year for the following decade, whereas MGUS progresses to active MM at a rate of about 1% per year [3,4].

Over the past two decades, the therapeutic landscape of MM has expanded substantially to include proteasome inhibitors, immunomodulatory agents, monoclonal antibodies, and alkylating agents. Despite the availability of several targeted therapies, the nitrogen mustard melphalan remains a clinically relevant component of MM treatment due to its well-established efficacy, manageable toxicity, and cost-effectiveness. Indeed, high-dose melphalan (HDM) followed by autologous stem cell transplantation (ASCT) remains the standard of care for transplant-eligible patients, while various combination regimens have been developed to improve efficacy and reduce toxicity [5]. Regardless of these advances and the resulting improvements in response rates and overall survival, MM remains largely incurable due to the emergence of drug resistance. Consequently, elucidating the molecular mechanisms that determine therapeutic response remains essential for improving long-term disease control [6,7,8].

Melphalan is an alkylating agent that induces multiple forms of DNA damage, including N-alkylpurine monoadducts, interstrand crosslinks (ICLs), and DNA–protein crosslinks [9]. Although ICLs represent a relatively small fraction of melphalan-induced lesions, they are considered cytotoxic because they block DNA replication and transcription, leading to replication fork collapse and the generation of DNA double-strand breaks (DSBs). DNA lesions are detected by specialized DNA damage response (DDR) sensor proteins, which activate appropriate repair pathways depending on the type of damage. N-alkylpurine monoadducts are repaired by nucleotide excision repair (NER), while the repair of ICLs is particularly complex and requires the coordinated action of multiple pathways, including the Fanconi anemia (FA) pathway, NER, base excision repair (BER), mismatch repair (MMR), and DSB repair mechanisms. DSBs are repaired via two pathways: homologous recombination (HR), which is mainly active during the S and G2 phases of the cell cycle, and non-homologous end joining (NHEJ), which operates throughout all phases of the cell cycle [2,10].

Beyond its direct genotoxic activity, melphalan also induces substantial oxidative stress through glutathione (GSH) depletion, increased production of reactive oxygen species (ROS), and lipid peroxidation. Elevated oxidative stress markers have been observed in MM patients, and HDM, used in conditioning for ASCT, induces further oxidative stress, contributing to treatment response and the development of drug resistance [7]. Importantly, restoration of intracellular GSH levels confers cytoprotection, alleviates cell cycle arrest, and enhances the survival of MM cells exposed to melphalan. Thus, modulation of redox status represents an additional mechanism by which MM cells may evade melphalan-induced cytotoxicity [8,11].

MM is characterized by marked genomic instability, including chromosomal translocations, copy number variations, and point mutations. Persistent DNA damage has been documented in MM cells, particularly in the form of DSBs, as demonstrated by constitutive phosphorylation of histone H2AX (γH2AX) [12]. Despite the presence of ongoing DNA damage, apoptotic responses are frequently attenuated due to loss-of-function mutations or deletions in key checkpoint regulators such as ataxia-telangiectasia mutated (ATM) and tumor protein p53 (TP53), as well as epigenetic silencing of TP73 [2,7,13].

Several DNA repair pathways influence cellular responses to melphalan and contribute to the development of drug resistance in MM. Alterations in single-strand break (SSB) repair have been reported, including overexpression of BER components such as APEX1 and APEX2 in MM cell lines and patient samples, which promotes melphalan resistance, together with dysregulation of HR-related genes such as RAD51 [14]. Similarly, variability in NER capacity has been linked to treatment response, with lower NER activity and slower DSB repair correlating with higher DNA damage accumulation and increased melphalan sensitivity [15]. Enhanced repair of DSBs and ICLs has also been observed in resistant MM cells, reflected by overexpression of FA/BRCA pathway components, which facilitates more efficient ICL removal [7]. Moreover, inherited polymorphisms in genes encoding components of HR, NER, and BER pathways have been associated with melphalan resistance [16]. Also, melphalan treatment itself may further exacerbate genomic instability by increasing the mutational burden in MM cells [2,17].

While considerable progress has been made in elucidating the roles of oxidative stress and dysregulated DNA repair pathways in melphalan resistance in multiple myeloma, it remains unclear whether baseline intrinsic differences in redox status and the DDR network can predict clinical sensitivity to genotoxic therapies. We hypothesized that primary cells from patients who subsequently respond to melphalan therapy exhibit a distinct functional profile, ultimately leading to enhanced apoptotic susceptibility following treatment. To test this hypothesis, we assessed DDR signals and redox status in peripheral blood mononuclear cells (PBMCs) and bone marrow plasma cells (BMPCs) obtained at baseline from multiple myeloma patients who were later classified as responders or non-responders to melphalan-based therapy.

## 2. Materials and Methods

### 2.1. Patients

BMPCs and PBMCs were obtained from seventy-six (*n* = 76) unselected newly diagnosed patients with MM (32F/44M; median age: 64.3 years; range: 44-81) (Table 1). Diagnosis was established according to the standard criteria of the International Myeloma Working Group (IMWG) [18], and patients were classified as responders (*n* = 35) or non-responders (*n* = 41) based on IMWG response criteria [19]. All patients’ samples were collected at diagnosis before treatment with any anti-myeloma or supportive treatment. All patients subsequently received HDM followed by ASCT. PBMCs were isolated as previously described [20]. Bone marrow aspirates were collected in EDTA-containing tubes, and mononuclear cells were separated using Ficoll-Paque density gradient centrifugation. CD138^+^ plasma cells were subsequently purified by positive magnetic-activated cell sorting (MACS) using immunomagnetic microbeads conjugated to an anti-CD138 monoclonal antibody (MACS CD138 MicroBeads, Miltenyi Biotec GmbH, Bergisch Gladbach, Germany). PBMCs and BMPCs were suspended in freezing medium [90% fetal bovine serum (FBS) and 10% dimethyl sulfoxide (DMSO)] and stored at −80 °C for up to 1 month prior to analysis. All samples underwent the same cryopreservation, storage, thawing, and analytical procedures. Samples were processed and analyzed in a blinded manner. Post-thaw cell viability was not systematically recorded. Primary cells were cultured in complete RPMI-1640 medium supplemented with 10% FBS, 100 units/mL penicillin, 100 μg/mL streptomycin, and 2 mmol/L L-glutamine. The study received approval from the Institutional Review Board of “Alexandra” Hospital and was carried out in accordance with the Declaration of Helsinki. Written informed consent was obtained from all participants.

### 2.2. Alkaline Comet Assay

The alkaline comet assay was performed according to the previously established protocol [21]. Briefly, 1 × 10^4^ cells were spread onto microscope slides in 1% low melting point agarose, lysed in alkaline lysis buffer (0.01M Tris, pH 10, 0.1M EDTA, 2.5M NaCl, 1% Triton X-100) for two hours at 4 °C, and electrophoresed for 30 min at 21 V, 300 mA, 4 °C. Slides were washed in water at 4 °C, fixed in ethanol (70%) and stained using SYBR™ Gold Nucleic Acid Gel Stain (Thermo Fisher Scientific, Waltham, MA, USA, #S11494). The Zeiss Axiophot (Zeiss, Oberkochen, Germany) fluorescence microscope was used for the visualization and photographic documentation of microscope slides. The Olive Tail Moment (OTM) parameter was calculated by CometScore free software v1.5 (TriTek Corp, Sumerduck, VA, USA). A minimum of 200 comets (cells) is scored per treatment group. Data are expressed as mean ± standard deviation (SD) based on at least three independent experiments.

### 2.3. Nucleotide Excision Repair Measurement

DNA repair capacity was assessed using the comet assay by measuring DNA damage induction and its subsequent repair over time. Repair efficiency was quantified based on the resolution of DNA strand breaks, visualized as comet tail formation. To evaluate the effectiveness of NER, cells were exposed to Ultraviolet C (UVC, 5 J/m^2^), incubated for 0–6 h at 37 °C in the proper medium, harvested, and subjected to alkaline comet assay [22]. It should be noted that the alkaline comet assay applied after UVC exposure does not directly quantify primary UVC-induced DNA photoproducts, but rather provides an indirect functional readout of NER activity by measuring strand breaks and alkali-labile sites generated during lesion processing.

### 2.4. Measurement of γH2AX Foci Formation/Removal

Cells were exposed to melphalan (100 μg/mL for 5 min), cultured for 0–48 h in drug-free medium, harvested, and aliquots containing 5 × 10^5^ cells were seeded onto poly-L-lysine–coated, UV-sterilized glass coverslips as described [23]. Following fixation with 4% paraformaldehyde for 15 min at room temperature (R/T), cells were permeabilized by 0.25% Triton X-100 in Phosphate-Buffered Saline (PBS; 2.7 mM KCl, 137 mM NaCl, 10 mM Na_2_HPO_4_, 1.8 mM KH_2_PO_4_, pH 7.4) for 10 min and blocked with 1% Bovine Serum Albumin (BSA) in PBS containing 0.25% Triton X-100 for 1 h at R/T. Samples were then incubated with anti-γH2AX primary antibody (Cell signaling, Danvers, MA, USA, #80312; 1:400 for 1 h at R/T), followed by an Alexa Fluor 488-conjugated secondary antibody (Invitrogen, Carlsbad, CA, USA, #481679; 1:1000) for 1 h at R/T in the dark. Coverslips were mounted onto glass slides using a DAPI-containing medium (EverBrite™ Hardset, Biotium, Fremont, CA, USA, #23004) and subsequently visualized and imaged using a Leica TCS SP-1 confocal microscope (Leica Microsystems, CMS GmbH, Mannheim, Germany). The γH2AX foci were manually scored in 100 cells per treatment, and results were expressed as the mean number of foci per nucleus ± standard deviation (SD) across three independent experiments.

### 2.5. Assessment of Chromatin Condensation

After being expanded for 30 min at 4 °C in hypotonic conditions (10mM Tris–HCl, pH 8.0, 10mM NaCl, 5mM MgCl_2_), cells were homogenized in 0.3% Nonidet P-40. Centrifugation (1500× *g* for 10 min) through a hypotonic buffer containing 8.5% sucrose was used to isolate nuclei, which were then resuspended in a digestion buffer. One unit of Micrococcal Nuclease (MNase; Takara Bio, San Jose, CA, USA, #2910A) was utilized to digest chromatin for 5 min at 37 °C. The reaction was stopped by adding an equivalent volume of stop solution (200 mM Tris–HCl, pH 8.0, 200 mM NaCl, 20 mM EDTA, 2% SDS, 200 μg/mL proteinase K). Purified genomic DNA was resolved on 1.5% agarose gel, moved to nitrocellulose membranes (Amersham Hybond-N+, Cytiva, Marlborough, MA, USA) and hybridized with N-ras–specific probes [24].

### 2.6. Oxidative Stress and Apurinic/Apyrimidinic Sites

A luminescence-based GSH/GSSG-Glo assay (Promega, Madison, WI, USA, V6612) was employed to evaluate oxidative stress by determining the ratio of reduced glutathione (GSH) to oxidized glutathione (GSSG). The OxiSelectTM Oxidative DNA Damage Quantification Kit (Cell Biolabs, San Diego, CA, USA, STA-324) was used to quantify apurinic/apyrimidinic (AP, abasic) sites. Every process was carried out in compliance with the manufacturer’s instructions.

### 2.7. Measurement of Apoptotic Sensitivity

Cells were exposed to escalating concentrations of melphalan for 5 min, followed by 24 h incubation in drug-free medium. Apoptotic sensitivity was then evaluated using the Cell Death Detection ELISAPLUS kit (Roche Diagnostics, Indianapolis, IN, USA; #11774425001), which quantitatively measures cytoplasmic histone-associated DNA fragments generated during apoptotic DNA fragmentation. Following cell lysis, apoptotic activity was determined by photometric measurement according to the manufacturer’s instructions. The assay provides a quantitative index of apoptotic DNA fragmentation and does not directly measure the percentage of apoptotic cells.

### 2.8. Expression of DDR-Associated Genes

Total RNA was extracted from BMPCs using the RNeasy Mini Kit (Qiagen, Frederick, MD, USA, #74104), according to the manufacturer’s instructions. The extracted RNA was stored at −80 °C until further use. The expression of 84 genes associated with DDR pathways was analyzed using the RT^2^ Profiler™ PCR Array (Qiagen, Frederick, MD, USA, PAHS-029Z). Differential gene expression was determined using the RT^2^ Profiler PCR Array Data Analysis Web Portal (https://geneglobe.qiagen.com/gr/analyze/), accessed on 3 August 2021.

### 2.9. Bioinformatic Analysis

Gene expression data derived from the RT^2^ Profiler PCR Array (QIAGEN) were analyzed using the ΔΔCt method [25]. Briefly, ΔCt values were calculated for each gene as the difference between the Ct of the gene of interest and the mean Ct of the reference genes (ACTB, B2M, GAPDH, HPRT1, and RPLP0). ΔΔCt values were then computed by comparing Responders to Non-Responders. Fold regulation (FR) was calculated as 2^(−ΔΔCt)^, and log_2_ fold change (log_2_FC) values were obtained as log_2_(FR), providing a symmetric scale for up- and down-regulation. Differentially expressed genes (DEGs) were defined using a combined threshold of fold regulation ≥ 1.5 and *p* ≤ 0.05. The use of a 1.5-fold change threshold was selected to balance sensitivity and biological relevance, allowing the inclusion of moderately regulated genes that may contribute to coordinated pathway-level effects. A more stringent cutoff (e.g., ≥2-fold) substantially reduced the number of detectable genes and limited downstream functional enrichment analysis. Bioinformatic and statistical analyses were performed in R (version 4.5.2; RStudio version 2026.01.1). Data visualization and analysis were conducted using the R packages ggplot2 [26], ComplexHeatmap [27], circlize, ggrepel, and dplyr. Functional enrichment analysis was performed using the enrichR v3.4 package [28] with the Reactome 2022 database [29]. Heatmaps were generated using normalized expression values (z-score transformation), and hierarchical clustering was performed using Euclidean distance and complete linkage. To account for multiple-hypothesis testing, *p*-values were additionally adjusted using the Benjamini–Hochberg false discovery rate (FDR) procedure.

### 2.10. Statistical Analysis

Continuous variables were tested for normal distribution by using the Kolmogorov–Smirnov test. Normally distributed data are presented as mean ± standard deviation, and comparisons between the two groups were performed using an independent *t*-test. Non-normally distributed data are presented as median (range), and comparisons between groups were performed using the Mann–Whitney U test. For DDR parameters correlation analysis, Pearson’s bivariate analysis was executed for primary cells. Finally, Principal Component Analysis (PCA) was conducted for clustering DDR parameters and investigating possible patterns among the parameters for primary cells. Hierarchical Clustering Analysis (HCA) was generated by using Z-score standardization of the DDR parameters and Ward’s Linkage to minimize variances. The SPSS software (for Windows, version 30.0, SPSS Inc., Chicago, IL, USA) was used for all calculations and performance of statistical analysis.

## 3. Results

### 3.1. DDR-Related Signals in Primary Cells from MM Patients

To test the hypothesis that primary cells from MM patients who eventually achieve a clinical response to melphalan display a unique functional phenotype that confers increased susceptibility to apoptosis upon drug exposure, PBMCs and BMPCs from 76 MM patients (35 responders and 41 non-responders to subsequent melphalan-based therapy) were analyzed at baseline.

**Figure 1:**
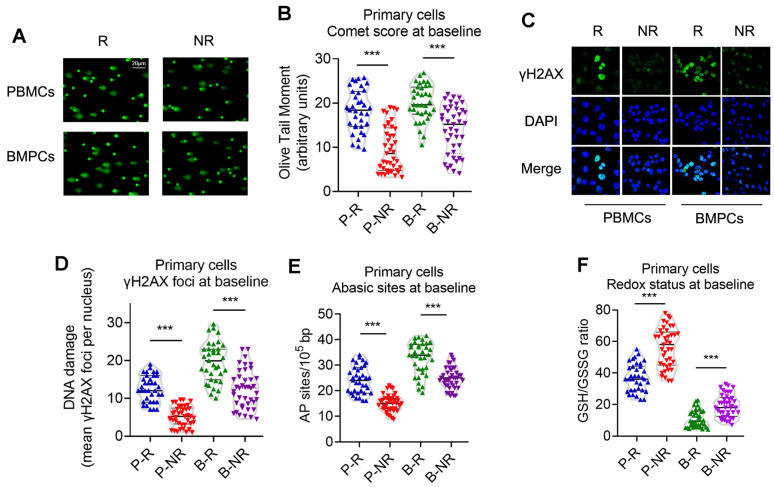
DDR parameters in primary cells at baseline. (**A**) Representative alkaline comet assay images of untreated PBMCs and BMPCs from one representative responder (R) and one non-responder (NR). Scale bar: 20 μm. (**B**) Baseline DNA strand breakage in PBMCs (P) and BMPCs (B) measured by comet assay. (**C**) Images showing γH2AX staining of untreated cells from one representative responder and one non-responder; upper images, γH2AX staining; middle, cell nuclei labelled with DAPI; bottom, merged. Magnification ×630. (**D**) Measurement of γH2AX foci in untreated cells. (**E**) Apurinic/apyrimidinic sites and (**F**) GSH/GSSG ratio in primary cells at baseline. Error bars indicate standard deviation (SD); *** *p* < 0.001.

First, using the alkaline comet assay, we found that untreated PBMCs and BMPCs from responders accumulated higher levels of DNA lesions (strand breaks and alkali-labile sites), compared with non-responders (*p* < 0.001; Table S1; Figure 1A,B). Next, we assessed additional markers associated with genomic stress and DDR activation. That is, γH2AX immunofluorescence was used as a surrogate marker of DSB-associated signaling/DDR activation, whereas abasic sites, a marker of DNA damage and genomic stress, were quantified using streptavidin-biotin interaction. Untreated PBMCs and BMPCs from responders exhibited higher levels of γH2AX foci and abasic lesions than the corresponding cells from non-responders (*p* < 0.001; Table S1; Figure 1C–E). Together with the elevated levels of strand breaks and alkali-labile sites detected by the alkaline comet assay, these findings indicate that responders display increased baseline genomic stress and DNA damage burden. To explore potential sources of the elevated baseline levels of strand breaks, alkali-labile lesions and abasic sites, we measured oxidative stress, which is known to contribute to the formation of such lesions [30]. We observed that untreated PBMCs and BMPCs from responders exhibited a decreased GSH-to-GSSG ratio, compared with non-responders (*p* < 0.001; Table S1; Figure 1F).

**Figure 2:**
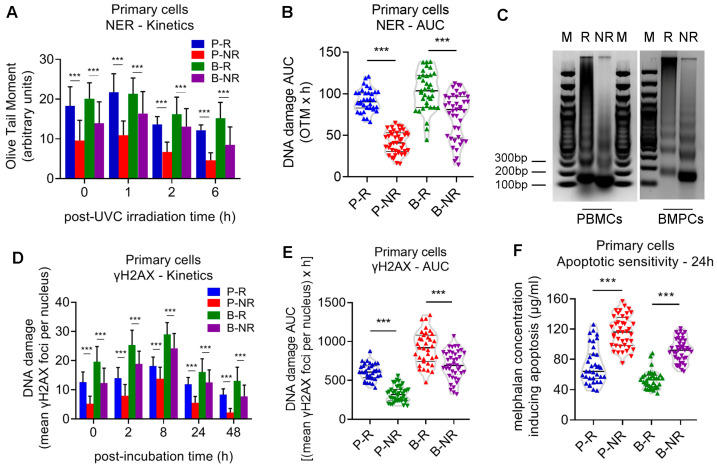
Nucleotide excision repair and γH2AX removal capacity of primary cells. (**A**) Kinetics of comet-detectable strand breaks and alkali-labile sites after UVC exposure, and (**B**) accumulation of these lesions, expressed as AUC, in primary cells after irradiation with 5 J/m^2^ UVC. (**C**) Chromatin condensation in PBMCs and BMPCs from one representative responder and one non-responder to melphalan therapy at baseline. M, 100 bp DNA ladder. The uncropped blots are shown in Figure S1. (**D**) Kinetics of γH2AX formation/removal, and (**E**) γH2AX burden, expressed as AUC, in primary cells following melphalan treatment. (**F**) Apoptotic sensitivity 24 h following melphalan treatment (0–200 μg/mL, 5min). Error bars represent SD; *** *p* < 0.001.

To further investigate cellular responses to UVC-induced DNA lesions, PBMCs and BMPCs were exposed to 5 J/m^2^ UVC, which induces the formation of 6-4 photoproducts (6-4PPs) and cyclobutane pyrimidine dimers (CPDs) [31]. The kinetics of comet-detectable strand breaks and alkali-labile sites were subsequently evaluated using the alkaline comet assay. In all individuals analyzed, maximal DNA damage levels were observed 1 h after UVC irradiation and subsequently declined. PBMCs and BMPCs from responders exhibited increased persistence of comet-detectable strand breaks and alkali-labile sites following UVC exposure compared with non-responders, suggesting differences in the cellular processing of UVC-induced DNA lesions (*p* < 0.001; Table S1; Figure 2A,B). Importantly, NER capacity, quantified as % repair, showed a pattern of differences between responders and non-responders in both PBMCs and BMPCs consistent with that observed in the AUC analysis, further supporting the robustness of the findings (Table S2).

Prior research has demonstrated that local chromatin structure significantly affects DNA repair activity and that DNA repair efficiency at the N-ras gene reflects the overall cellular NER capacity [24,32]. In both PBMCs and BMPCs, following micrococcal nuclease digestion, the N-ras gene gave rise to higher-order chromatin structures in primary cells from responders, whereas the same gene gave rise predominantly to mono- and di-nucleosome structures in the corresponding cells from non-responders (Figure 2C and Figure S1).

Next, PBMCs and BMPCs were treated with 100 μg/mL melphalan for 5 min, and γH2AX foci were quantified by confocal microscopy to assess the kinetics of DSB-associated DDR signal resolution. Primary cells exhibited maximal γH2AX levels 8 h after melphalan treatment, decreasing thereafter (Figure 2D). Responders exhibited prolonged persistence of melphalan-induced γH2AX foci compared with non-responders, consistent with differences in the kinetics of DSB-associated DDR signal resolution following melphalan exposure (*p* < 0.001; Table S1; Figure 2D,E). Notably, γH2AX foci removal capacity, expressed as % removal, yielded results comparable to those obtained using the AUC approach in both PBMCs and BMPCs, further supporting the observed differences in DNA repair capacity between responders and non-responders (Table S2).

Apoptotic sensitivity was also evaluated in both PBMCs and BMPCs twenty-four hours following melphalan exposure. We found that primary cells from non-responders required higher concentrations of melphalan to induce apoptotic DNA fragmentation, suggesting significantly reduced apoptotic sensitivity compared with responders (*p* < 0.001; Table S1; Figure 2F).

### 3.2. Expression of DDR-Associated Genes in BMPCs from MM Patients

**Figure 3:**
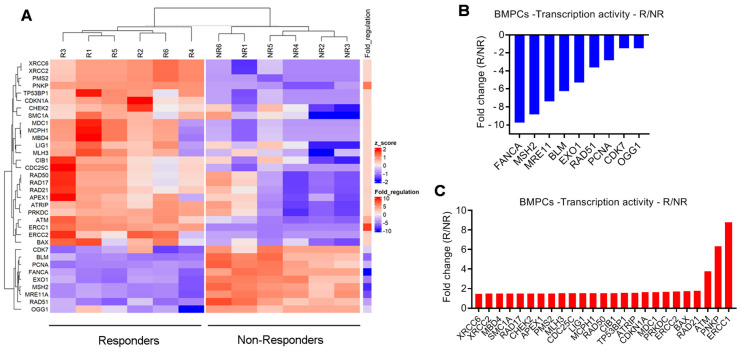
Gene expression analysis of DDR-associated genes. (**A**) Heat map of 34 differentially expressed genes in BMPCs from 12 MM patients (6 responders versus 6 non-responders). (**B**,**C**) Genes demonstrating at least 1.5-fold difference in the transcription activity between responders and non-responders.

To further investigate the deregulated DDR network in MM, the expression of 84 DDR-associated genes was analyzed in BMPCs from 12 MM patients at baseline (6 responders and 6 non-responders) (Table S3). A total of 34 differentially expressed genes, representing several non-mutually exclusive categories, exhibited at least a 1.5-fold difference in expression between responders and non-responders, with 25 genes being overexpressed and 9 genes being downregulated (Figure 3 and Figure S2).

In addition, we have performed multiple-testing correction across the entire DDR-gene panel using the Benjamini–Hochberg false discovery rate (FDR) procedure. The corresponding FDR-adjusted *p*-values have been included in Table S4. Because the study was based on a targeted panel of DDR genes and focused on pathway-level analysis, we retained the predefined biologically relevant threshold for downstream functional enrichment, while providing the adjusted *p*-values (FDR) for transparency (Table S5). Nine genes that were found downregulated in responders versus non-responders were categorized into DSB repair (RAD51, BLM, MRE11), ICL repair (FANCA), MMR (EXO1, MSH2), and NER (OGG1, CDK7, PCNA) pathways (Table S5). Moreover, twenty-five genes that were overexpressed in responders versus non-responders were sub-categorized into seven groups as follows: (a) genes involved in DSB repair (ATM, PNKP, RAD21, PRKDC, TP53BP1, RAD50, XRCC2, XRCC6), (b) NER-related genes (ERCC1, ERCC2, LIG1), (c) genes involved in BER (APEX1, MBD4), (d) cell cycle-associated genes (MDC1, CDKN1A, ATRIP, MCPH1, CDC25C, PMS2, CHEK2), (e) apoptosis-related genes (BAX, CIB1), (f) signaling genes (RAD17, SMC1A), and g) MMR genes (MLH3) (Table S5).

Next, to identify the most significantly altered DDR-related processes, pathway enrichment analysis was performed using Enrichr. Statistical significance (*p* < 0.05) was observed in several DDR pathways (Figures S3 and S4), including DNA Repair (*p* = 2.30 × 10^−19^), DNA Double-Strand Break Repair (*p* = 2.89 × 10^−13^), HDR through Homologous Recombination (HRR) or Single-Strand Annealing (SSA) (*p* = 5.54 × 10^−11^), HDR through Single Strand Annealing (SSA) (*p* = 7.19 × 10^−11^), Homology-Directed Repair (*p* = 9.35 × 10^−11^), Homologous DNA Pairing and Strand Exchange (*p* = 2.61 × 10^−10^), G2/M DNA damage checkpoint regulation (*p* = 4.69 × 10^−10^), and HDR through Homologous Recombination (HRR) (*p* = 4.78 × 10^−10^).

### 3.3. Statistical Analysis of DDR Parameters

Statistical analysis of the seven DDR parameters under study was conducted in primary cells obtained from MM patients. Pearson correlation analysis is shown in Table 2. All DDR parameters revealed significant correlations among them for both PBMCs and BMPCs. Correlation analysis for PBMCs showed moderate to very strong positive associations for all DDR parameters except Apoptotic sensitivity, which was associated negatively with Baseline AP-Sites, Baseline DNA damage, NER (AUC), Baseline γH2AX foci and γH2AX foci (AUC) but positively with Baseline GSH/GSSG Ratio. Same results were observed for BMPCs, with correlations ranging from weak to very strong.

**Figure 4:**
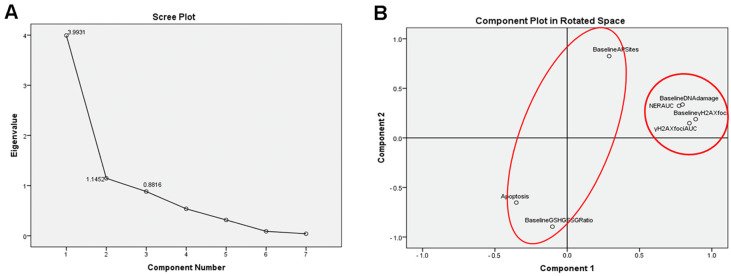
Statistical analysis in BMPCs. (**A**) Scree plot from Principal Component Analysis (PCA). (**B**) Component matrix after varimax rotation.

In BMPCs, Bartlett’s Test of sphericity (*p* < 0.001) and KMO (0.714) indicated that PCA was statistically reliable and interpretable. The eigenvalues of the component matrix are depicted in Figure 4A. PCA pointed out a two-factor solution explaining 73.4% of the variance. Varimax rotation was applied, and results for BMPCs are shown in Table S6 and illustrated in Figure 4B. The first component comprised Baseline γH2AX foci, γH2AX foci (AUC), Baseline DNA damage and NER (AUC), suggesting that the loadings positively affect the direction of the model. On the other hand, the second component comprised Baseline GSH/GSSG Ratio, Baseline AP-Sites and Apoptotic sensitivity. The first and the last loading were negative, as previously mentioned in the correlation matrix.

**Figure 5:**
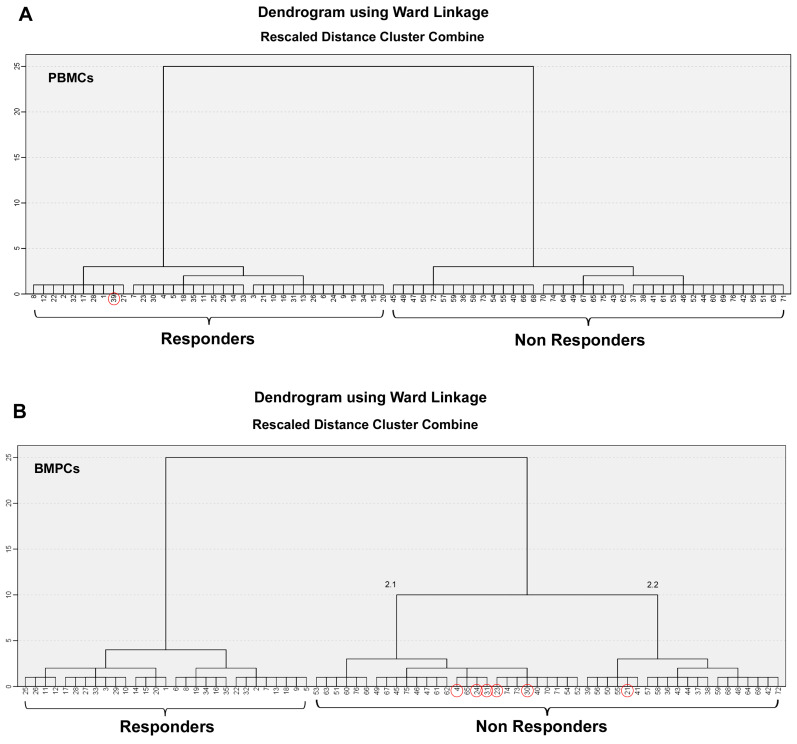
Statistical analysis of DDR parameters for primary cells. Dendrograms of PBMCs (**A**) and BMPCs (**B**) after application of HCA. As 2.1 was named the first cluster and 2.2 the second cluster of the second branching.

Principal Component Analysis of PBMC samples did not reveal a clear separation between the study groups, as the first principal components explained the major proportion of variance without demonstrating distinct group-specific clustering. In contrast, Hierarchical Clustering Analysis identified clustering patterns among the cases, suggesting similarities in PBMC profiles that were not captured by the PCA model. For the purposes of HCA and to improve sample identification, responders and non-responders were assigned values from 1 to 76. The responder group comprised cases 1 to 35, while the non-responder group included cases 36 to 76. Primary taxonomic separation in PBMC samples, after applying HCA, demonstrated a high degree of homogeneity and clear separation according to therapy response, with nearly all responders clustered together (except for case 39, which was a non-responder), while the remaining non-responders formed a second cluster (Figure 5A). Overall, the distinct clustering of the groups at the first major branching indicates that DDR parameters of redox status, DNA damage, apoptotic sensitivity, and DNA repair mechanisms in blood samples were effective and efficient classifiers for distinguishing responders from non-responders to therapy.

Data derived from BMPCs were more complex. Repeatedly, HCA revealed two distinct phenotypes in relation to response and non-response to therapy (Figure 5B). Cluster 1 represented a highly homogeneous group of patients who responded to therapy, although not in their entirety, as six cases clustered within the second group. These results suggest that the DDR parameters were able to characterize a distinct profile for most patients who responded to therapy. The second cluster was more heterogeneous. It predominantly consisted of non-responders but also included a small number of responders (cases 4, 21, 23, 24, 30, and 31). Within the intra-subgroup structure of this cluster, subcluster 2.1 included five responders (cases 4, 24, 31, 23, and 30), while subcluster 2.2 included case 21. This internal variance may suggest a potential two-subphenotype structure of non-responsiveness to therapy based on the measured DDR parameters.

Collectively, consistent patterns in redox status, DNA damage, DNA repair capacity (including nucleotide excision repair [NER] and double-strand break [DSB] repair), and apoptotic response were observed in both BMPCs and PBMCs, effectively distinguishing responders from non-responders. These findings further support our initial hypothesis that baseline redox status and DDR-related pathways assessed at diagnosis are associated with subsequent clinical outcomes.

## 4. Discussion

DNA damage response is a sophisticated, evolutionarily conserved signaling network that detects DNA damage and activates repair mechanisms, cell cycle checkpoints, or apoptosis to preserve genome stability. Because cancer cells often have altered or deficient DDR pathways, chemotherapy takes advantage of these vulnerabilities by causing excessive, irreparable DNA damage that ultimately triggers cell cycle arrest and apoptosis. Based on this rationale, we sought to elucidate the association between DNA repair efficiency, redox status and response to melphalan-based therapy in MM. Importantly, the study was designed as a retrospective analysis, in which patients were categorized based on their subsequent clinical response to melphalan-based therapy. Therefore, the primary aim was to identify associations between baseline biological features and treatment outcome rather than to establish predictive biomarkers. Accordingly, the observed molecular differences should be interpreted as potential response-associated signatures that may provide biological insight into treatment heterogeneity in MM.

DNA damage constitutes a constant threat to cellular integrity, as it may lead to mutagenesis, genomic instability, and apoptosis [33]. Understanding both the levels and types of DNA damage, as well as the mechanisms driving their accumulation, is essential for elucidating the molecular basis of drug resistance. In the present study, responders to subsequent anti-myeloma therapy exhibited higher levels of baseline DNA damage than non-responders. These findings are consistent with a central principle in oncology: tumors with high baseline DNA damage, often resulting from increased DNA lesion formation or impaired DNA repair, tend to be more susceptible to DNA-damaging therapy [34,35,36].

To further investigate the mechanisms underlying baseline DNA damage in MM patients, we examined oxidative stress, a key factor influencing chemotherapy sensitivity and a well-established source of DNA damage [37]. We found that responders exhibited lower GSH-to-GSSG ratios in both BMPCs and PBMCs compared with non-responders. Increased oxidative stress has previously been reported in MM and has been proposed as a contributing factor to disease pathogenesis [23]. The high immunoglobulin production characteristic of MM is thought to contribute significantly to ROS overproduction [38]. The elevated oxidative stress observed in responders may also partly explain the increased levels of apurinic/apyrimidinic sites detected in these patients, since ROS are known to induce such DNA lesions [37].

To explore the contribution of DNA repair mechanisms to the accumulation of endogenous DNA damage, comet-detectable strand breaks and alkali-labile sites generated following UVC exposure were monitored over time using the alkaline comet assay. Importantly, this assay provides an indirect functional readout of NER–associated processing rather than a direct measurement of UVC-induced photoproduct removal. Therefore, the observed differences likely reflect variations in lesion processing efficiency rather than direct quantification of primary UVC lesion excision. We found that BMPCs and PBMCs from non-responders exhibited a faster decline in comet-detectable strand breaks and alkali-labile sites following UVC exposure compared with responders, suggesting differences in cellular responses to UVC-induced DNA lesions. These findings are consistent with previous reports indicating that NER capacity in MM cells strongly influences response to therapeutic agents, particularly alkylating drugs such as melphalan, by determining the ability of plasma cells to repair drug-induced DNA damage [15,39,40]. Notably, high expression of the NER-related gene ERCC3, which encodes a subunit of the transcription factor TFIIH, has been associated with poor overall survival in patients treated with high-dose melphalan. Consistent with this observation, inhibition of the NER pathway through targeting the XPB helicase (encoded by ERCC3) using compounds such as spironolactone or triptolide significantly increases the sensitivity of MM cells to alkylating agents and may overcome drug resistance [15,41].

Chromatin condensation was also examined. We observed that responders displayed more condensed chromatin in malignant BMPCs compared with non-responders, a pattern similarly reflected in PBMCs. These observations are consistent with previous studies indicating that chromatin structure plays a complex role in chemotherapy response, as the degree of chromatin compaction influences DNA accessibility and thereby affects both the binding of chemotherapeutic agents to DNA and the efficiency of DNA repair processes [23,42,43]. In fact, compact heterochromatin, often located at the nuclear periphery, restricts the access of chemotherapeutic agents to the DNA, thus contributing to drug resistance [44]. In addition, tightly packed chromatin hinders the recruitment of DNA repair machinery to lesions. Consequently, heterochromatic regions often exhibit higher mutation rates due to reduced repair efficiency [45]. To overcome this, cells actively remodel chromatin at the site of damage, increasing local accessibility. This involves histone modifications (e.g., H4K16 acetylation) and the action of ATP-dependent remodeling complexes, such as SWI/SNF, which evict nucleosomes to allow access [46].

Many cytotoxic agents, including melphalan, platinum-based drugs, and topoisomerase inhibitors such as etoposide, act by inducing DNA DSBs. Cancer cells that efficiently cope with DSB-induced damage may survive treatment, leading to therapy failure and disease recurrence [47,48,49,50]. In line with this concept, our analysis showed that non-responders exhibited faster removal of melphalan-induced γH2AX foci and reduced persistence of the associated DDR signal compared with responders in both BMPCs and PBMCs. Previous studies have demonstrated that elevated HR activity in MM contributes to genomic instability, progressive accumulation of loss of heterozygosity (LOH), and resistance to dexamethasone [51]. Moreover, deregulated NHEJ efficiency has been associated with increased MM risk and poor prognosis [52].

We also examined the expression of DDR-related genes in BMPCs at baseline. ATM, ERCC1, and PNKP were upregulated in responders compared with non-responders, whereas BLM, EXO1, FANCA, MRE11A, MSH2, PCNA, and RAD51 were downregulated. Previous studies have linked increased ERCC1 expression with improved clinical outcomes following treatment with dexamethasone or thalidomide [53], while higher PCNA expression in bone marrow biopsies correlates with advanced MM stage [54]. In addition, BLM mutations have been associated with the t(11;14) translocation in MM [55], and high BLM expression has been linked to shorter overall survival [56]. Increased RAD51 expression has also been reported in melphalan-resistant myeloma cell lines [57], while upregulation of FANCA and the Fanconi anemia pathway has been observed in melphalan-resistant cells [40]. Notably, seven of the genes identified here as downregulated in responders are among the seventeen DNA repair genes previously reported to be associated with poor prognosis in MM, whereas three of the genes upregulated in responders belong to the subset linked to favorable prognosis [58].

The differences observed between responders and non-responders may reflect the considerable genetic and epigenetic heterogeneity that characterizes ΜΜ. Genomic alterations, including chromosomal abnormalities, copy number changes, clonal evolution, and mutations affecting DDR pathways, may influence genomic stability, DNA repair capacity, and susceptibility to melphalan-induced damage. Likewise, epigenetic mechanisms such as DNA methylation, histone modifications, chromatin remodeling, and non-coding RNA regulation can modulate the expression of genes involved in DNA repair, oxidative stress responses, and apoptosis. Together, these molecular features may contribute to the distinct redox status, chromatin organization, and DNA repair profiles observed in this study. Further genomic and epigenomic investigations will be required to elucidate the mechanisms underlying these differences [2,59,60,61,62].

In line with previous reports [23,63,64], our findings revealed significantly reduced apoptotic sensitivity in primary cells derived from non-responders compared with responders. These findings further support the notion that intrinsic redox status, differences in DNA damage processing, and altered DNA damage response signaling kinetics contribute to treatment sensitivity and clinical outcome.

Several limitations of this study should be acknowledged. First, the retrospective design and post hoc classification of patients into responder and non-responder groups limit the interpretation of the findings to associative rather than predictive relationships. In addition, the gene expression analyses were performed in a relatively small cohort and should therefore be considered exploratory. The limited sample size also precluded robust multivariable analyses incorporating established prognostic factors, such as ISS stage and cytogenetic abnormalities. Consequently, the independent prognostic and predictive value of the identified DDR-related biomarkers could not be determined and requires validation in larger, prospectively collected cohorts with comprehensive clinical and molecular annotation. Furthermore, the observed associations between oxidative stress, DNA damage accumulation, chromatin organization, DNA repair capacity, and treatment response are correlative and do not establish causality. Although the findings support a biologically plausible mechanistic framework, functional studies employing targeted modulation of oxidative stress and DNA repair pathways will be required to define the causal relationships underlying melphalan sensitivity and resistance. Also, chromatin condensation was assessed primarily through the qualitative evaluation of micrococcal nuclease digestion patterns. Although consistent differences were observed between responders and non-responders, quantitative measurements of chromatin accessibility were not conducted. Future studies incorporating comprehensive chromatin accessibility assays, such as ATAC-seq, together with histone modification profiling, may provide further validation of these findings and offer additional mechanistic insights. In addition, the functional assays used in this study provided a global assessment of DDR activity but did not directly evaluate specific DNA repair pathways or checkpoint signaling mechanisms. Finally, the differentially expressed DDR-related genes were not independently validated at the mRNA or protein level. Future studies incorporating pathway-specific analyses and molecular validation approaches, including RAD51 and 53BP1 foci analyses, neutral comet assays, cell-cycle profiling, and assessment of checkpoint activation markers, will be important to further elucidate the mechanisms underlying differential treatment responses in MM.

A key finding of our study is that tumor characteristics may be reflected in readily accessible peripheral blood cells, supporting the concept that circulating immune cells can serve as a minimally invasive window into tumor-associated biological processes. This phenomenon is increasingly recognized as a consequence of bidirectional communication between malignant cells, the tumor microenvironment, and the systemic immune compartment [65]. In ΜΜ, immune cells undergo functional reprogramming through direct cell–cell interactions, inflammatory signaling, exposure to tumor-derived soluble mediators, and extracellular vesicles that transfer proteins, lipids, and nucleic acids capable of modulating immune-cell gene expression and function [65,66,67]. In parallel, chronic inflammation, oxidative stress-related systemic effects, immune dysregulation, and cancer-associated epigenetic alterations, including DNA methylation changes and chromatin remodeling, may further shape PBMC transcriptional profiles [66,68]. Collectively, these mechanisms suggest that PBMCs may capture both direct tumor–immune interactions and broader systemic effects of malignancy. However, the relative contribution of these processes remains unclear and warrants further investigation.

## 5. Conclusions

**Figure 6:**
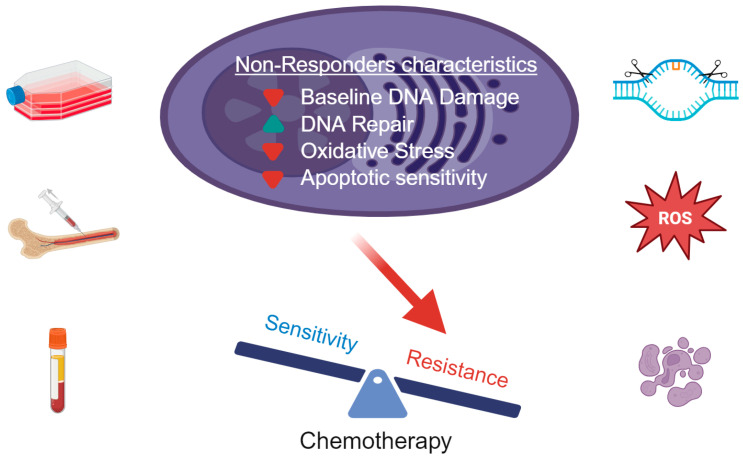
DNA damage response-related parameters potentially underlying resistance to alkylating therapy in MM, including enhanced DNA repair capacity, lower levels of baseline DNA damage and oxidative stress, and reduced apoptotic sensitivity. Created in Biorender. Malamos P. (2026) https://BioRender.com/6r9llm6 (accessed on 14 June 2026).

Taken together, these findings suggest that malignant plasma cells from MM patients who subsequently did not respond to alkylating drug therapy may exhibit a distinct molecular profile, characterized by lower baseline levels of DNA damage and oxidative stress, enhanced DNA repair capacity, and reduced apoptotic activity compared with those from responders (Figure 6). Notably, PBMCs from the same patients also displayed significant differences in redox status and DDR-related signals between the two clinical outcome groups, suggesting that peripheral blood cells may reflect systemic disease-associated biological alterations. However, the clinical relevance of these observations remains to be established. Prospective studies in well-characterized, independent patient cohorts will be required to validate these findings and to determine whether the observed DDR-, redox-, and apoptosis-related differences can be translated into clinically useful predictive biomarkers.

## Figures and Tables

**Table 1:** Patient and disease characteristics.

Characteristic	No. of Patients (% of Total)
**Gender**	
Female	32 (42.1%)
Male	44 (57.9%)
**Age (years)**	
Median	64.3
Range	44 to 81
**Serum M-protein isotype**	
*Heavy chain*	
IgA	21 (27.6%)
IgG	43 (56.5%)
IgM	1 (1.3%)
None detected	11 (14.4%)
*Light chain*	
Kappa light chain	51 (67.1%)
Lambda light chain	23 (30.3%)
None detected	2 (2.6%)
**ISS stage**	
I	31 (40.1%)
II	26 (34.2%)
III	19 (25.0%)
**Cytogenetic risk** ^a^	
Standard	59 (77.6%)
High	17 (22.4%)
**Response to HDM**	
Responders	35 (46.1%)
Non-responders	41 (53.9%)

Ig, immunoglobulin; ISS, International Staging System; HDM, High-dose melphalan; ^a^ the cytogenetic risk was defined as high based on the presence of del(17p), translocation t(4;14), and/or translocation t(14;16).

**Table 2:** Pearson correlation analysis among DDR parameters for PBMCs and BMPCs.

PBMCs						
	Baseline GSH/GSSG Ratio	Baseline AP-Sites	Baseline DNA Damage	NER (AUC)	Baseline γH2AX Foci	γH2AX Foci (AUC)
**Baseline AP-Sites**	−0.647 **					
**Baseline DNA damage**	−0.622 **	0.542 **				
**NER (AUC)**	−0.684 **	0.663 **	0.901 **			
**Baseline γH2AX foci**	−0.563 **	0.495 **	0.660 **	0.776 **		
**γH2AX foci (AUC)**	−0.578 **	0.590 **	0.614 **	0.787 **	0.842 **	
**Apoptotic sensitivity**	0.578 **	−0.551 **	−0.553 **	−0.667 **	−0.600 **	−0.652 **
**BMPCs**						
**Baseline AP-Sites**	−0.670 **					
**Baseline DNA damage**	−0.372 **	0.470 **				
**NER (AUC)**	−0.361 **	0.453 **	0.955 **			
**Baseline γH2AX foci**	−0.288 *	0.437 **	0.591 **	0.564 **		
**γH2AX foci (AUC)**	−0.278 *	0.401 **	0.513 **	0.459 **	0.906 **	
**Apoptotic sensitivity**	0.478 **	−0.503 **	−0.419 **	−0.382 **	−0.471 **	−0.403 **

** Correlation is significant at the 0.01 level (2-tailed). * Correlation is significant at the 0.05 level (2-tailed).

## Data Availability

The original contributions presented in this study are included in the article/Supplementary Materials. Further inquiries can be directed to the corresponding author.

## Supplementary Materials

The following supporting information can be downloaded at: https://www.mdpi.com/article/10.3390/cancers18121995/s1, Table S1. Comparison of DDR parameters for responders and non-responders in PBMCs and BMPCs; Table S2. DNA repair capacity (% repair) according to treatment response; Table S3. RT^2^ Profiler™ PCR Array Human DNA Damage Signaling Pathway: Gene list; Table S4. Results of multiple-testing correction across the DDR gene panel using the Benjamini–Hochberg FDR procedure; Table S5. Differentially expressed genes (DEGs): fold regulation ≥ 1.5 and *p*-value ≤ 0.05. Adjusted *p*-values (FDR) are also provided; Table S6. Rotated component matrix and factor loading after varimax rotation in BMPCs; Figure S1. Uncropped Southern blot images corresponding to the data presented in Figure 2C; Figure S2. Volcano plot of differentially expressed genes (DEGs) between responders and non-responders; Figure S3. Dot plot showing the top 20 Reactome pathways enriched among the differentially expressed genes (DEGs), ranked by statistical significance; Figure S4. Mapping of 34 differentially expressed genes to the Top 20 enriched Reactome Pathways.
