# Supplementary material for: Breaking the Balance: Baseline Oxidative Stress and DNA Repair Capacity in Multiple Myeloma Therapy
Source: Cancers (Basel). 2026 Jun 19;18(12):1995. doi: 10.3390/cancers18121995 (PMC13297473; doi:10.3390/cancers18121995)
Supplement: Supplementary file 1 [file cancers-18-01995-s001.zip › cancers-4341350-supplementary.pdf]

Supplementary Materials

Breaking the Balance: Baseline Oxidative Stress and DNA Repair Capacity in Multiple Myeloma Therapy

Malamos P. et al.

Table of Contents

- Table S1. Comparison of DDR parameters for responders and non-responders in PBMCs and BMPCs
- Table S2. DNA repair capacity (% repair) according to treatment response
- Table S3. RT² Profiler™ PCR Array Human DNA Damage Signaling Pathway: Gene list
- Table S4. Results of multiple-testing correction across the DDR gene panel using the Benjamini–Hochberg FDR procedure
- Table S5. Differentially expressed genes (DEGs): fold regulation ≥ 1.5 and *p*-value ≤ 0.05. Adjusted *p*-values (FDR) are also provided
- Table S6. Rotated component matrix and factor loading after varimax rotation in BMPCs
- Figure S1. Uncropped Southern blot images corresponding to the data presented in Figure 2C
- Figure S2. Volcano plot of differentially expressed genes (DEGs) between responders and non-responders
- Figure S3. Dot plot showing the top 20 Reactome pathways enriched among the differentially expressed genes (DEGs), ranked by statistical significance
- Figure S4. Mapping of 34 differentially expressed genes to the Top 20 enriched Reactome Pathways

Table S1. Comparison of DDR parameters for responders and non-responders in PBMCs and BMPCs

	**Responders**	**Non-responders**	***p*-value** [a]
	**PBMCs**		
**Baseline DNA Damage**	18.3 (±4.8)	9.6 (±5.1)	< 0.001
**Baseline γH2AX foci**	12.6 (±3.5)	5.2 (±2.6)	< 0.001
**Baseline AP-Sites**	24.1 (±5.3)	15.1 (±3.3)	< 0.001
**Baseline GSH/GSSG Ratio**	37.1 (±8.9)	56.2 (±13)	< 0.001
**NER (AUC)**	91.8 (±13.6)	41.5 (±13.4)	< 0.001
**γH2AX foci (AUC)**	608.5 (±103.4)	324.2 (±100.6)	< 0.001
**Apoptotic sensitivity**	72.3 (±24.5)	116.7 (±21)	< 0.001
	**BMPCs**		
**Baseline DNA Damage**	20.08 (± 4.03)	13.91 (± 5.42)	< 0.001
**Baseline γH2AX foci**	19.62 (±5.24)	12.35 (± 5.05)	< 0.001
**Baseline AP-Sites**	32.73 (± 6.31)	24.81 (± 4.17)	< 0.001
**Baseline GSH/GSSG Ratio**	10.79 (± 5.41)	19.15 (± 7.23)	< 0.001
**NER (AUC)**	102.63 (± 23.5)	73.23 (± 27.71)	< 0.001
**γH2AX foci (AUC)**	915.35 (±214.23)	696.48 (± 182.56)	< 0.001
**Apoptotic sensitivity**	53.06 (± 11.82)	91.83 (± 15.5)	< 0.001

[a] t-test, values are presented as mean ± SD

Table S2. DNA repair capacity (% repair) according to treatment response

DDR marker	Cell type	Responders	Non Responders	*p* value
**NER capacity**[a]	**PBMC**	44.8 (18.1, 15.3 to 60.5)[c]	50.5 (19.0, 36.1 to 90.0)[c]	< 0.001[e]
	**BMPC**	28.8 (13.1, 13.0 to 64.2)[c]	47.0 (29.0, 29.5 to 93.3)[c]	< 0.001[e]
**γH2AX removal capacity**[b]	**PBMC**	53.2 (18.3, 28.1 to 78.3)[c]	84.7 (9.0, 58.2 to 94.1)[c]	< 0.001[e]
	**BMPC**	56.4 (11.2, 40.7 to 80.2)[d]	68.5 (14.4, 41.3 to 99.1)[d]	< 0.001[f]

[a] NER capacity (% repair) was calculated as the percentage reduction in the mean number of Olive Tail Moment (arbitrary units) from the peak level observed at 1h after irradiation to the final measurement at 6h; [b] γH2AX removal capacity (% removal) was calculated as the percentage reduction in the mean number of γH2AX foci per cell from the peak level observed at 8h after treatment to the final measurement at 48h; [c] values are presented as median (IQR, min to max); [d] values are presented as mean (± SD, min to max); [e] Mann-Whitney test; [f] independent t-test

Table S3. RT[2] Profiler™ PCR Array Human DNA Damage Signaling Pathway: Gene list

No	Symbol	GeneBank	Description
1	ABL1	NM_005157	C-abl oncogene 1, non-receptor tyrosine kinase
2	APEX1	NM_080649	APEX nuclease (multifunctional DNA repair enzyme) 1
3	ATM	NM_000051	Ataxia telangiectasia mutated
4	ATR	NM_001184	Ataxia telangiectasia and Rad3 related
5	ATRIP	NM_032166	ATR interacting protein
6	ATRX	NM_000489	Alpha thalassemia/mental retardation syndrome X-linked
7	BARD1	NM_000465	BRCA1 associated RING domain 1
8	BAX	NM_004324	BCL2-associated X protein
9	BBC3	NM_014417	BCL2 binding component 3
10	BLM	NM_000057	Bloom syndrome, RecQ helicase-like
11	BRCA1	NM_007294	Breast cancer 1, early onset
12	BRIP1	NM_032043	BRCA1 interacting protein C-terminal helicase 1
13	CDC25A	NM_001789	Cell division cycle 25 homolog A (S. pombe)
14	CDC25C	NM_001790	Cell division cycle 25 homolog C (S. pombe)

15	CDK7	NM_001799	Cyclin-dependent kinase 7
16	CDKN1A	NM_000389	Cyclin-dependent kinase inhibitor 1A (p21, Cip1)
17	CHEK1	NM_001274	CHK1 checkpoint homolog (S. pombe)
18	CHEK2	NM_007194	CHK2 checkpoint homolog (S. pombe)
19	CIB1	NM_006384	Calcium and integrin binding 1 (calmyrin)
20	CRY1	NM_004075	Cryptochrome 1 (photolyase-like)
21	CSNK2A2	NM_001896	Casein kinase 2, alpha prime polypeptide
22	DDB1	NM_001923	Damage-specific DNA binding protein 1, 127kDa
23	DDB2	NM_000107	Damage-specific DNA binding protein 2, 48kDa
24	DDIT3	NM_004083	DNA-damage-inducible transcript 3
25	ERCC1	NM_001983	Excision repair cross-complementing rodent repair deficiency, complementation group 1 (includes overlapping antisense sequence)
26	ERCC2	NM_000400	Excision repair cross-complementing rodent repair deficiency, complementation group 2
27	EXO1	NM_130398	Exonuclease 1
28	FANCA	NM_000135	Fanconi anemia, complementation group A
29	FANCD2	NM_033084	Fanconi anemia, complementation group D2
30	FANCG	NM_004629	Fanconi anemia, complementation group G
31	FEN1	NM_004111	Flap structure-specific endonuclease 1
32	GADD45A	NM_001924	Growth arrest and DNA-damage-inducible, alpha
33	GADD45G	NM_006705	Growth arrest and DNA-damage-inducible, gamma
34	H2AFX	NM_002105	H2A histone family, member X
35	HUS1	NM_004507	HUS1 checkpoint homolog (S. pombe)
36	LIG1	NM_000234	Ligase I, DNA, ATP-dependent
37	MAPK12	NM_002969	Mitogen-activated protein kinase 12
38	MBD4	NM_003925	Methyl-CpG binding domain protein 4
39	MCPH1	NM_024596	Microcephalin 1
40	MDC1	NM_014641	Mediator of DNA-damage checkpoint 1
41	MLH1	NM_000249	MutL homolog 1, colon cancer, nonpolyposis type 2 (E. coli)
42	MLH3	NM_014381	MutL homolog 3 (E. coli)
43	MPG	NM_002434	N-methylpurine-DNA glycosylase

44	MRE11A	NM_005590	MRE11 meiotic recombination 11 homolog A (S. cerevisiae)
45	MSH2	NM_000251	MutS homolog 2, colon cancer, nonpolyposis type 1 (E. coli)
46	MSH3	NM_002439	MutS homolog 3 (E. coli)
47	NBN	NM_002485	Nibrin
48	NTHL1	NM_002528	Nth endonuclease III-like 1 (E. coli)
49	OGG1	NM_002542	8-oxoguanine DNA glycosylase
50	PARP1	NM_001618	Poly (ADP-ribose) polymerase 1
51	PCNA	NM_182649	Proliferating cell nuclear antigen
52	PMS1	NM_000534	PMS1 postmeiotic segregation increased 1 (S. cerevisiae)
53	PMS2	NM_000535	PMS2 postmeiotic segregation increased 2 (S. cerevisiae)
54	PNKP	NM_007254	Polynucleotide kinase 3'-phosphatase
55	PPM1D	NM_003620	Protein phosphatase, Mg2+/Mn2+ dependent, 1D
56	PPP1R15A	NM_014330	Protein phosphatase 1, regulatory (inhibitor) subunit 15A
57	PRKDC	NM_006904	Protein kinase, DNA-activated, catalytic polypeptide
58	RAD1	NM_002853	RAD1 homolog (S. pombe)
59	RAD17	NM_002873	RAD17 homolog (S. pombe)
60	RAD18	NM_020165	RAD18 homolog (S. cerevisiae)
61	RAD21	NM_006265	RAD21 homolog (S. pombe)
62	RAD50	NM_005732	RAD50 homolog (S. cerevisiae)
63	RAD51	NM_002875	RAD51 homolog (S. cerevisiae)
64	RAD51B	NM_133509	RAD51 homolog B (S. cerevisiae)
65	RAD9A	NM_004584	RAD9 homolog A (S. pombe)
66	RBBP8	NM_002894	Retinoblastoma binding protein 8
67	REV1	NM_016316	REV1 homolog (S. cerevisiae)
68	RNF168	NM_152617	Ring finger protein 168
69	RNF8	NM_183078	Ring finger protein 8
70	RPA1	NM_002945	Replication protein A1, 70kDa
71	SIRT1	NM_012238	Sirtuin 1

72	SMC1A	NM_006306	Structural maintenance of chromosomes 1A
73	SUMO1	NM_003352	SMT3 suppressor of mif two 3 homolog 1 (S. cerevisiae)
74	TOPBP1	NM_007027	Topoisomerase (DNA) II binding protein 1
75	TP53	NM_000546	Tumor protein p53
76	TP53BP1	NM_005657	Tumor protein p53 binding protein 1
77	TP73	NM_005427	Tumor protein p73
78	UNG	NM_003362	Uracil-DNA glycosylase
79	XPA	NM_000380	Xeroderma pigmentosum, complementation group A
80	XPC	NM_004628	Xeroderma pigmentosum, complementation group C
81	XRCC1	NM_006297	X-ray repair complementing defective repair in Chinese hamster cells 1
82	XRCC2	NM_005431	X-ray repair complementing defective repair in Chinese hamster cells 2
83	XRCC3	NM_005432	X-ray repair complementing defective repair in Chinese hamster cells 3
84	XRCC6	NM_001469	X-ray repair complementing defective repair in Chinese hamster cells 6

Table S4. Results of multiple-testing correction across the DDR gene panel using the Benjamini–Hochberg FDR procedure

**No**	**Gene**	**log2FC**	***p* value**	**FDR**
1	ABL1	-0.506556645	0.07027	0.11387
2	APEX1	0.590676706	0.02574	0.05941
3	ATM	1.916599385	0.00002	0.00013
4	ATR	0.045266273	0.89137	0.89137
5	ATRIP	0.659799454	0.00869	0.02275
6	ATRX	-0.215020481	0.41631	0.46834

7	BARD1	-0.269696722	0.21941	0.27481
8	BAX	0.796783447	0.00539	0.01673
9	BBC3	-0.420076346	0.19295	0.25167
10	BLM	-2.647601269	0	0.00002
11	BRCA1	-0.324445066	0.26392	0.30788
12	BRIP1	0.366782531	0.21664	0.27481
13	CDC25A	-0.327072159	0.26683	0.30788
14	CDC25C	0.612329622	0.00209	0.00819
15	CDK7	-0.586930204	0.0012	0.00514
16	CDKN1A	0.694544356	0.00035	0.00167
17	CHEK1	-0.197348063	0.1638	0.21679
18	CHEK2	0.581126771	0.03411	0.07139
19	CIB1	0.625752858	0.02973	0.0637
20	CRY1	-0.395990168	0.14006	0.19392
21	CSNK2A2	0.490349933	0.07528	0.11681
22	DDB1	0.128899926	0.61644	0.67658
23	DDB2	0.518217718	0.06233	0.10788
24	DDIT3	0.481706849	0.00695	0.01979
25	ERCC1	3.132313187	0	0
26	ERCC2	0.770685278	0.00116	0.00514
27	EXO1	-2.408459508	0	0.00002
28	FANCA	-3.285669943	0	0

29	FANCD2	-0.469093993	0.0211	0.05276
30	FANCG	0.330213151	0.09324	0.13757
31	FEN1	0.554470054	0.07079	0.11387
32	GADD45A	0.544988073	0.07499	0.11681
33	GADD45G	-0.357450067	0.22887	0.27836
34	H2AFX	0.476658127	0.11822	0.16625
35	HUS1	-0.563667102	0.07085	0.11387
36	LIG1	0.616796508	0.05142	0.09641
37	MAPK12	-0.436718204	0.02922	0.0637
38	MBD4	0.571734707	0.0071	0.01979
39	MCPH1	0.624227703	0.00424	0.01467
40	MDC1	0.708166392	0.0019	0.00777
41	MLH1	-0.204101225	0.25647	0.30372
42	MLH3	0.605837897	0.02541	0.05941
43	MPG	0.10050015	0.72515	0.76781
44	MRE11A	-2.88412248	0	0
45	MSH2	-3.146684518	0	0.00001
46	MSH3	0.50011249	0.05552	0.09994
47	NBN	-0.428483502	0.09313	0.13757
48	NTHL1	-0.532414976	0.04379	0.08568
49	OGG1	-0.584006531	0.00516	0.01659
50	PARP1	0.53142965	0.00885	0.02275

51	PCNA	-1.490787068	0	0.00002
52	PMS1	-0.242663229	0.00033	0.00163
53	PMS2	0.599298587	0	0
54	PNKP	2.665.521.224	0	0
55	PPM1D	-0.410469022	0	0.00003
56	PPP1R15A	0.407201043	0	0.00003
57	PRKDC	0.738888908	0.00488	0.01626
58	RAD1	-0.319406628	0.2229	0.27481
59	RAD17	0.579913915	0.03999	0.07997
60	RAD18	-0.229392468	0.37263	0.42452
61	RAD21	0.830403102	0.00702	0.01979
62	RAD50	0.626164889	0.02898	0.0637
63	RAD51	-1.858831174	0.00003	0.00016
64	RAD51B	0.469097196	0.09607	0.13946
65	RAD9A	0.584599849	0.06413	0.1089
66	RBBP8	-0.353007057	0.23753	0.28504
67	REV1	0.497137608	0.10726	0.15323
68	RNF168	0.446115883	0.14342	0.19392
69	RNF8	-0.591808401	0.06142	0.10788
70	RPA1	-0.550625285	0.07847	0.1197
71	SIRT1	0.332939841	0.22122	0.27481
72	SMC1A	0.574042663	0.04822	0.09233

73	SUMO1	-0.466354976	0.00224	0.00823
74	TOPBP1	0.302822683	0.02456	0.05941
75	TP53	-0.46498428	0.00229	0.00823
76	TP53BP1	0.648034826	0.00021	0.00111
77	TP73	-0.278622156	0.03527	0.07215
78	UNG	0.03014248	0.87894	0.88881
79	XPA	-0.210930126	0.00726	0.01979
80	XPC	-0.071451998	0.66523	0.72133
81	XRCC1	-0.136824815	0.05435	0.09983
82	XRCC2	0.56425009	0	0.00003
83	XRCC3	-0.428438533	0.00005	0.00026
84	XRCC6	0.539267338	0.00001	0.00004
85	ACTB	0.099445714	0.14436	0.19392
86	B2M	0.014588571	0.71805	0.76781
87	GAPDH	-0.011982857	0.76654	0.78396
88	HPRT1	0.013731429	0.73387	0.768
89	RPLP0	-0.115782857	0.45587	0.50652
90	HGDC	0.05516	0.74357	0.76921

Table S5. Differentially expressed genes (DEGs): fold regulation ≥ 1.5 and *p*-value ≤ 0.05. Adjusted *p*-values (FDR) are also provided

No	Symbol	*p*-value	Fold regulation	FDR	DDR pathway involved
1	ERCC1	0.000	8,768	7.57E-08	NER
2	PNKP	0.000	6,345	2.48E-06	DSB/R (NHEJ)
3	ATM	0.000	3,775	0.000125357	DSB/R (HR)
4	RAD21	0.007	1,778	0.019787468	DSB/R
5	BAX	0.005	1,737	0.016731869	Apoptosis
6	ERCC2	0.001	1,706	0.005136459	NER
7	PRKDC	0.005	1,669	0.016258313	DSB/R, Apoptosis
8	MDC1	0.002	1,634	0.007771786	Cell cycle
9	CDKN1A	0.000	1,618	0.001672659	Cell cycle, Apoptosis
10	ATRIP	0.009	1.580	0.022745744	Cell cycle, Signaling
11	TP53BP1	0.000	1,567	0.001111495	DSB/R
12	RAD50	0.029	1,543	0.063701359	DSB/R
13	CIB1	0.030	1,543	0.063701359	Apoptosis
14	MCPH1	0.004	1,541	0.014674386	Cell cycle
15	LIG1	0.051	1,533	0.096413933	NER, BER, DSB/R
16	CDC25C	0.002	1,529	0.008186482	Cell cycle
17	MLH3	0.025	1,522	0.059408268	MMR
18	PMS2	0.000	1,508	2.68E-06	Cell cycle
19	APEX1	0.025	1,506	0.059408268	BER
20	CHEK2	0.034	1,496	0.071394252	Cell cycle
21	RAD17	0.040	1,495	0.07997247	Signaling
22	SMC1A	0.048	1,489	0.092330967	Signaling

23	MBD4	0.007	1,486	0.019787468	BER
24	XRCC2	0.000	1,479	0.0000321	DSB/R
25	XRCC6	0.000	1,453	4.36E-05	DSB/R
26	OGG1	0.000	−1,499	0.016590497	NER, BER
27	CDK7	0.000	−1,502	0.005136459	NER, Cell cycle
28	PCNA	0.000	−2,81	1.94E-05	NER, MMR, BER
29	RAD51	0.000	−3,627	0.0001602	DSB/R
30	EXO1	0.000	−5,309	0.0000218	DSB/R (HR), MMR
31	BLM	0.000	−6,266	0.0000218	DSB/R (HR)
32	MRE11	0.000	−7,383	0.0000036	DSB/R
33	MSH2	0.001	−8,856	0.00000756	MMR
34	FANCA	0.005	−9,752	2.68E-06	ICL/R

Table S6. Rotated component matrix and factor loading after varimax rotation in BMPCs

	**Component**	
	**1**	**2**
**Baseline γH2AX foci**	0.888	
**γH2AX foci (AUC)**	0.845	
**Baseline DNA damage**	0.797	
**NER (AUC)**	0.773	
**Baseline GSH/GSSG Ratio**		−0.896
**Baseline AP-Sites**		0.825
**Apoptotic sensitivity**		−0.653

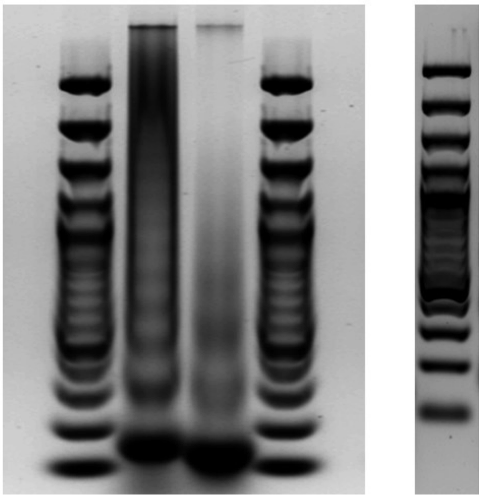

Figure S1. Uncropped Southern blot images corresponding to the data presented in Figure 2C. Representative images showing chromatin condensation in PBMCs and BMPCs from one responder and one non-responder to melphalan therapy at baseline. Molecular weight markers are also shown. The uncropped images are provided to document the integrity of the original experimental data.

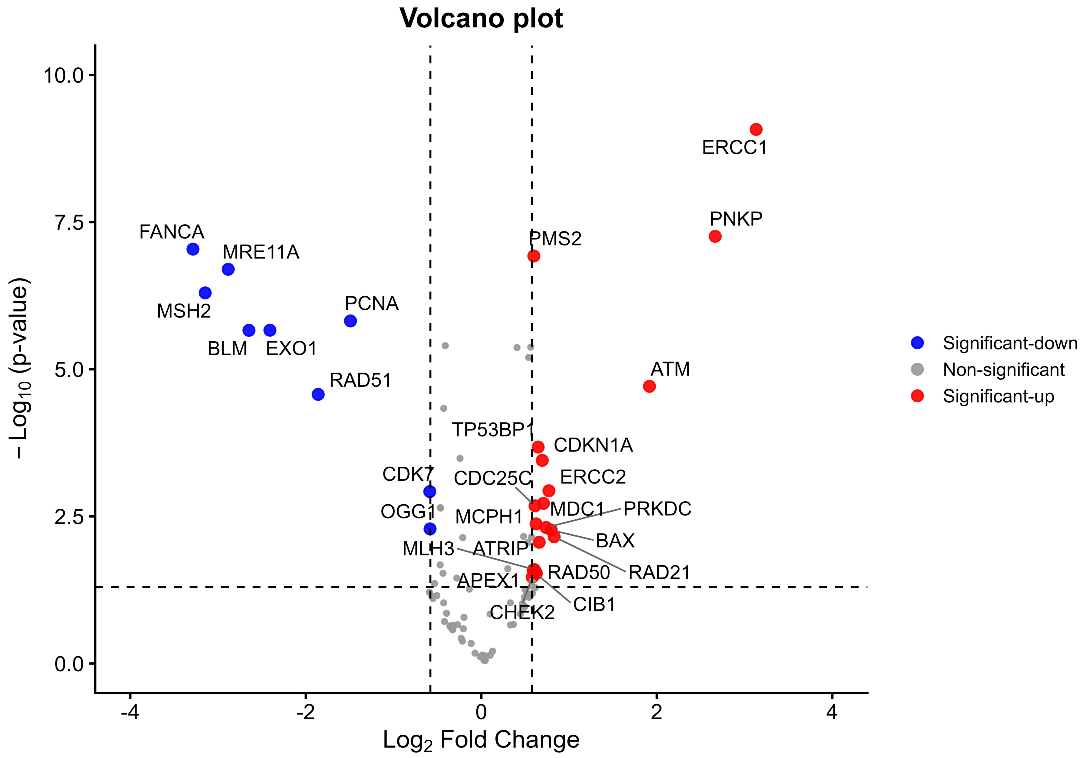

Figure S2. Volcano plot of differentially expressed genes (DEGs) between responders and non-responders. Differential gene expression profiling was performed using the RT² Profiler PCR Array (QIAGEN), targeting 90 genes related to DDR pathways, including five housekeeping genes used for normalization. Differentially expressed genes between responders and non-responders were identified using a threshold of fold regulation ≥ 1.5 (log2FC ≥ 0.58) and $p \leq 0.05$.The x-axis represents the $\log_2$ fold change (responders versus. non-responders), while the y-axis shows the $-\log_{10}$(p-value). Vertical dashed lines indicate the fold change threshold (± 0.58 in $\log_2$ scale), and the horizontal dashed line represents the statistical significance threshold ($p = 0.05$). Significantly upregulated genes (Significant-up) in responders are shown in red, whereas significantly downregulated genes (Significant-down) are shown in blue. Non-significant genes (Non-significant) are depicted in grey.

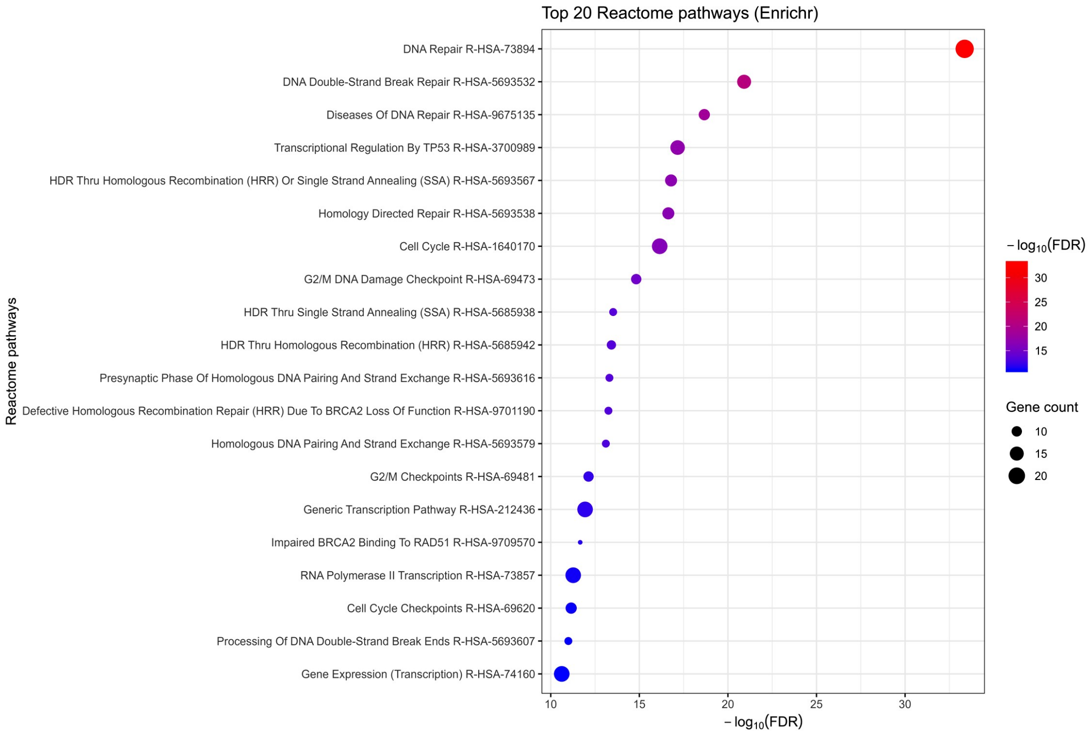

Figure S3. Dot plot showing the top 20 Reactome pathways enriched among the differentially expressed genes (DEGs), ranked by statistical significance. Dot size corresponds to the number of DEGs associated with each pathway, and color intensity reflects the level of statistical significance ($-\log_{10}$(FDR)), with higher values indicating stronger enrichment.

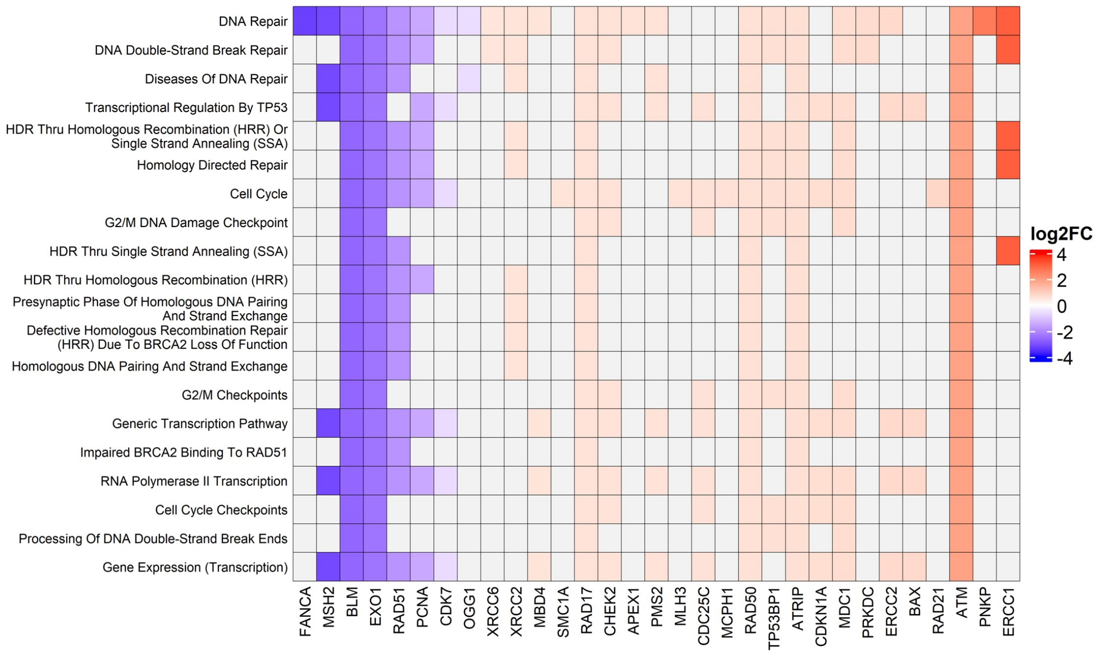

Figure S4. Mapping of 34 differentially expressed genes to the Top 20 enriched Reactome Pathways. Color scale was based on the log2FC of DEGs between responders and non-responders.
